# Interlaboratory Reproducibility in Growth and Reporter Expression in the Cyanobacterium *Synechocystis* sp. PCC 6803

**DOI:** 10.1021/acssynbio.3c00150

**Published:** 2023-05-29

**Authors:** Maurice Mager, Hugo Pineda Hernandez, Fabian Brandenburg, Luis López-Maury, Alistair J. McCormick, Dennis J. Nürnberg, Tim Orthwein, David A. Russo, Angelo Joshua Victoria, Xiaoran Wang, Julie A. Z. Zedler, Filipe Branco dos Santos, Nicolas M. Schmelling

**Affiliations:** † Institute for Synthetic Microbiology, Heinrich Heine University Duesseldorf, Universitaetsstrasse 1, 40225 Duesseldorf, Germany; ‡ Molecular Microbial Physiology Group, Swammerdam Institute for Life Sciences, Faculty of Science, University of Amsterdam, Science Park 904, Amsterdam 1098 XH, The Netherlands; § Helmholtz Centre for Environmental Research (UFZ), Permoserstrasse 15, 04318 Leipzig, Germany; ∥ Instituto de Bioquímica Vegetal y Fotosíntesis, University of Seville − CSIC, Américo Vespucio 49, 41092 Sevilla, Spain; ⊥ Departamento de Bioquímica Vegetal y Biología Molecular, Facultad de Biología, University of Seville, Avenida Reina Mercedes, 41012 Sevilla, Spain; # Institute of Molecular Plant Sciences, School of Biological Sciences, University of Edinburgh, 1.04 Daniel Rutherford Building, King’s Buildings, EH9 3BF Edinburgh, U.K.; ∇ Department of Physics, Experimental Biophysics, Freie University Berlin, Arnimallee 14, 14195 Berlin, Germany; ■ Dahlem Centre of Plant Sciences, Freie Universität Berlin, Albrecht-Thaer-Weg 6, 14195 Berlin, Germany; ⊗ Interfaculty Institute of Microbiology and Infection Medicine, University of Tuebingen, Auf der Morgenstelle 28, 72076 Tübingen, Germany; □ Institute for Inorganic and Analytical Chemistry, Bioorganic Analytics, Friedrich Schiller University Jena, Lessingstrasse 8, 07743 Jena, Germany; ○ Matthias Schleiden Institute for Genetics, Bioinformatics and Molecular Botany, Synthetic Biology of Photosynthetic Organisms, Friedrich Schiller University Jena, Dornburgerstrasse 159, 07743 Jena, Germany

**Keywords:** promoter, cyanobacteria, reproducibility, interlab

## Abstract

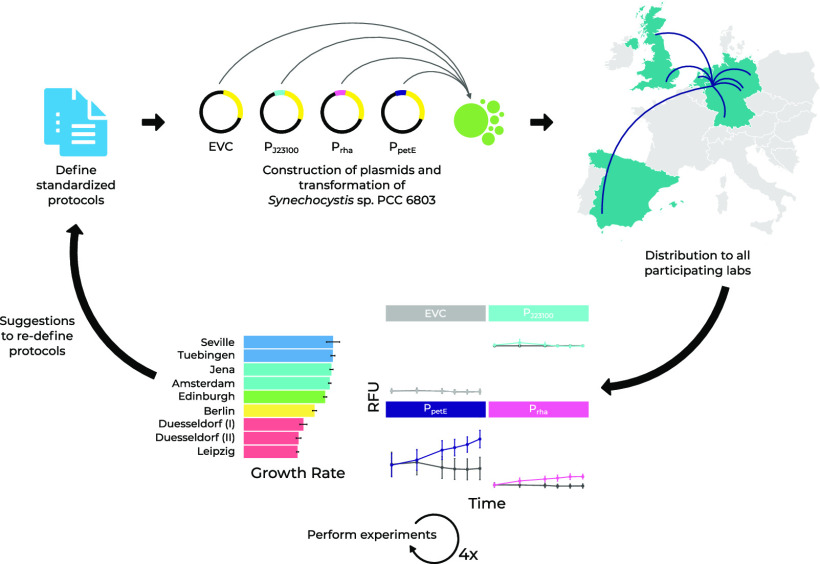

In recent years, a plethora of new synthetic biology tools for use in cyanobacteria have been published; however, their reported characterizations often cannot be reproduced, greatly limiting the comparability of results and hindering their applicability. In this interlaboratory study, the reproducibility of a standard microbiological experiment for the cyanobacterial model organism *Synechocystis* sp. PCC 6803 was assessed. Participants from eight different laboratories quantified the fluorescence intensity of mVENUS as a proxy for the transcription activity of the three promoters P_*J*23100_, P_*rhaBAD*_, and P_*petE*_ over time. In addition, growth rates were measured to compare growth conditions between laboratories. By establishing strict and standardized laboratory protocols, reflecting frequently reported methods, we aimed to identify issues with state-of-the-art procedures and assess their effect on reproducibility. Significant differences in spectrophotometer measurements across laboratories from identical samples were found, suggesting that commonly used reporting practices of optical density values need to be supplemented by cell count or biomass measurements. Further, despite standardized light intensity in the incubators, significantly different growth rates between incubators used in this study were observed, highlighting the need for additional reporting requirements of growth conditions for phototrophic organisms beyond the light intensity and CO_2_ supply. Despite the use of a regulatory system orthogonal to *Synechocystis* sp. PCC 6803, P_*rhaBAD*_, and a high level of protocol standardization, ∼32% variation in promoter activity under induced conditions was found across laboratories, suggesting that the reproducibility of other data in the field of cyanobacteria might be affected similarly.

## Introduction

As oxygenic phototrophs, cyanobacteria can potentially mitigate climate change when utilized by a carbon-neutral biotechnological industry. However, robust molecular biology tools are needed to study their physiology, enable their manipulation, and tap into their potential as green biotechnology platforms. Over the past decades, a plethora of new molecular tools like molecular manipulation tools and CRISPR/Cas systems have been developed for various model species.^1−3^ Those tools have been adapted and further extended for cyanobacteria at an unprecedented rate in recent years. RSF1010-based replicative plasmid for various cyanobacterial species^4^ and chromosomal integration for introducing transgenes have been used for decades to introduce heterologous genes into cyanobacteria. Many constitutive and inducible promoters as well as novel terminators have been characterized for transgenic expression in cyanobacteria.^5−7^ Inducible promoters have been successfully implemented in more sophisticated applications such as CRISPR and CRISPRi systems as well as for metabolic engineering.^3,8^ However, to ensure a seamless transfer of inducible promoter tools to other investigators, their performance needs to be optimally characterized under different experimental conditions to give users a sense of their robustness and limitations.

Usually, those promoters are described and tested in cyanobacterial model strains like *Synechocystis* sp. PCC 6803 (hereafter PCC 6803) or *Synechococcus elongatus* PCC 7942. However, Cyanobacteria are a diverse phylum, represented by multiple model strains, which show differences in their optimal cultivation conditions, their ability for genetic manipulation, and the behavior of genetic parts. While *lacI*-based inducible expression systems have been commonly used in *Synechococcus*(9) and *Anabaena*,^10^ attempts to design similar systems for PCC 6803 showed very little tunability and high leaky expression.^11^ Further, different investigators have reported extreme variability in promoter activity among PCC 6803 substrains. Reported fold changes for those inducible promoters range from 3- to 32-fold for the P_*petE*_ promoter and 30- to 55-fold for variations on the P_*rhaBAD*_ promoter in PCC 6803^3,6,12^ (see Table S1). For the P_*petE*_ promoter, varying basal activity levels have been reported^13−15^ (see Table S2). Due to the lack of a standardized measurement unit for promoter activity, comparisons among different reports in the literature are nearly impossible. Thus, despite the efforts to create robust genetic tools for cyanobacteria, the implementation of inducible promoters often requires readaptation of the promoter sequence and sometimes also the protocols, even when used in the same cyanobacterial model strain under seemingly identical cultivation conditions.

Reproducibility of research results has been noted as a problem in various fields and dubbed as the Reproducibility Crisis, including improper documentation of equipment and detailed methods.^16^ This problem is much more extensive for cyanobacterial research as culture conditions for phototrophs are very complex. Variations in light intensity and quality, as well as CO_2_ availability in different incubators, are additional factors that influence growth rates and cellular metabolism. Furthermore, it has been shown that optical density (OD), a standard reporting unit for the growth of bacteria, is not comparable across devices, strains, and, thus, laboratories.^17^ This fact is especially an issue when using dose-responsive inducible promoters, as the ratio of inducer molecules to the number of cells will determine the actual induction rather than a defined inducer concentration.^6^ However, OD is still the most dominant form used to report bacterial growth and define the starting point for experiments.

Further, the respective mRNA sequence can influence the observed promoter activity,^18^ as well as the type of assay used to measure promoter activity, such as fluorescence or luminescence-based reporters, protein quantification, or Northern blot. All these factors lead to different reports on promoter strengths and fold changes of inducible promoters.

Interlaboratory comparisons (ILCs) are, according to the European Commission Science Hub, organized to either “check the ability of laboratories to deliver accurate testing results” or to “find out whether a certain analytical method performs well and is fit for its intended purposes”.^19^ Within an ILC, the same analytical method is performed by multiple laboratories on the same samples. Subsequently, the results of each independent laboratory are compared in terms of conformity and deviation. As such, ILCs are used to assess each laboratory’s accuracy and the method’s accuracy in general. The term “interlab study” was recently popularized within the synthetic biology community by the international Genetically Engineered Machines (iGEM) competition as an ILC study to assess the reproducibility of properties from single genetic elements inside a defined context.^20−23^ Such studies are a valuable tool to investigate the robustness of methods and expression platforms and identify possible sources of variation. However, those studies are seldom reported and, to our knowledge, have not been performed with cyanobacteria to test the reproducibility of widely used techniques such as promoter activity or growth.

We, therefore, set up an interlab study in eight different laboratories to investigate the reproducibility of a relatively simple growth and promoter activity quantification experiment in the cyanobacterial model strain PCC 6803. We designed a protocol reflecting state-of-the-art methods reported in the literature and performed the experiment four times in each laboratory. Three commonly used promoters for heterologous expression in PCC 6803, P_*J*23100_, P_*petE*_, and P_*rhaBAD*_ were chosen, and their transcriptional activity was quantified using the fluorescent reporter protein mVENUS as a proxy. While actual transcript levels and to a lesser extent protein levels may fluctuate in response to minute changes in environmental conditions, we chose mVENUS expression as a simplified form to approximate transcript and resulting protein levels, which we further refer to as “mVENUS expression”. Experimental conditions were standardized, leaving equipment and investigators as the primary sources of variability. OD measurements were highly reproducible within replications in a single laboratoy. However, we noticed that dilutions of the initially identical cultures to the starting OD resulted in vastly different cell biomass concentrations across laboratories. This effect results from taking OD values as a proxy for cell concentration instead of cell count. In the following, we observed significant differences in growth rate across laboratories that could not be found to correlate with the initial difference in cell biomass concentration. Even when expression of the promoters of interest was induced with concentrations above saturation level, high variability of promoter strengths was observed across different laboratories. With this information, we aim to formulate best practices for reporting these parameters to ensure better reproducibility and robustness of research results in the future.

## Results and Discussion

The aim of this interlab study was to assess the reproducibility of routinely performed microbiological experiments for the cyanobacterial model organism PCC 6803. The experiment of choice was a time series of the transcription strength of three promoters in PCC 6803 using the fluorescence reporter mVENUS, representing common procedures used frequently in molecular biology laboratories. Those experiments include the growth of PCC 6803 under “standard” cultivation conditions and fluorescence measurements in a plate reader and should not require overly sophisticated equipment. Furthermore, the flask type as well as the flask cap, light intensity, and shaking speed were defined to reduce the number of confounding factors. Thus, we performed the same predefined experiments (see Methods) under conditions as identical as possible across all eight independent laboratories routinely working with cyanobacteria. This study is hence aimed to give a broad, unbiased picture of the current state of reproducibility of some of the published methods in cyanobacterial research.

### Selection and Design of Genetic Parts and Constructs

Two widely used inducible promoters have been chosen as representative candidates. The rhamnose-dependent *rhaBAD* system is based on the *E. coli* native *rhaBAD* operon regulated by the AraC-like positive transcription regulator RhaS. Upon addition of rhamnose, RhaS dimers bind to the RhaS regulon and recruit RNA polymerases by interaction with *E. coli* sigma 70 factor RpoD.^24^ Based on sequence similarity, it has been hypothesized that in PCC 6803, RhaS recruits the main sigma factor SigA instead. This is further supported by conserved sites between RpoD and SigA, which were previously described as relevant for RhaS-RpoD interaction.^13^ RhaS-based regulation of the *rhaBAD* promoter was utilized in PCC 6803 to reach a 55× induction when RhaS was expressed from a strong constitutive promoter J23119.^6^

The copper-dependent *petE* system is based on the PCC 6803 native promoter of the *petE* gene, which is responsible for plastocyanin expression. The *petE* promoter is regulated by an interplay of PetR and PetP: PetR represses transcription of *petE* by binding to the *petE* promoter, while PetP is a protease that degrades PetR in the presence of copper.^14^ Englund et al. observed a 5× induction of the *petE* promoter in PCC 6803 in the presence of copper^15^ using the same experimental approach as the one used in this manuscript.

The J23100 promoter is a constitutive promoter from the Anderson promoter collection.^25^ This collection is a small combinatorial library of J23119 derivatives and covers multiple orders of magnitude in transcription strength in *E. coli*. The entire collection has been characterized in PCC 6803 by Vasudevan et al.^5^

Each individual promoter was cloned upstream of the bicistronic design (BCD2) ribosomal binding site (RBS).^25^ This module consists of a ribosomal binding site followed by a short reading frame, a stop codon, and a secondary ribosomal binding site. Through translational coupling, this sequence has been reported to minimize coding sequence-based bias to translation activity. Additionally, the insulation effect of the BCD2 ribosomal binding site reduces background activity from upstream genes, improving the fold changes of inducible promoter systems. We chose this RBS to reduce deviation through genetic design further. In addition, each individual promoter was cloned upstream of the *mVENUS* coding sequence on the RSF1010 origin of replication, a commonly used shuttle vector for PCC 6803.

As an empty vector control (hereafter: EVC), the RSF1010 background only harboring the chloramphenicol resistance gene was used. A single designated laboratory was chosen to clone the desired constructs. Further, a defined PCC 6803 background strain was selected to mitigate any effects that different PCC 6803 background strains might have on the results. The designated laboratory transformed the PCC 6803 background strain and later shipped cryopreserved stock cultures on dry ice to each participating laboratory to ensure that each laboratory had genetically identical strains for the experiments.

### Experimental Setup of the Interlab Study

We created a set of detailed protocols to standardize the experimental conditions and data collection across all participants. The protocols contained the required information to handle the (frozen) stocks of the strains, prepare the growth medium, set up the incubator conditions, handle the cultures during the experiment, and perform the measurements.

In short, glycerol stocks of the four strains, EVC, P_*J*23100_, P_*petE*_, and P_*rhaBAD*_ (Table 1), were inoculated in liquid culture and grown for 36 to 48 h. The day before the assay, each preculture was diluted to OD_730_ of 0.3 to ensure cells grew exponentially at the onset of the assay. On the next day, each culture was divided into two flasks (induced and uninduced), the OD_730_ was adjusted to 0.5 when necessary, and inducers were added accordingly. From this moment, samples were taken during the first seven hours and after 24 h (see Figure S1 for a graphical representation of the protocol). In these samples, growth was recorded by measuring OD_730_ in a benchtop spectrophotometer and promoter activity by measuring fluorescence and OD_730_ in a plate reader. Additionally, full absorption spectra were measured as a control for the vitality of the cultures and as an internal quality control. However, these data were not included in this analysis because no notable changes in absorption spectra were observed. The raw data can be found at 10.6084/m9.figshare.21525747.v5.

**Table 1:** Strains Used in This Study

strain name	fluorescent reporter	reporter promoter	inducer
EVC	–	–	–
P_*J*23100_	mVENUS	P_*J*23100_	constitutive
P_*petE*_	mVENUS	P_*petE*_	CuSO_4_
P_*rhaBAD*_	mVENUS	P_*rhaBAD*_	rhamnose

### OD_730_ Measurements Are Highly Reproducible

**Figure 1:**
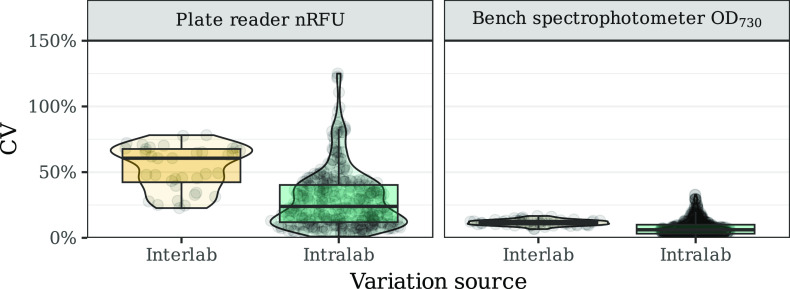
Coefficient of variation (%) of nRFU in EVC, P_*rhaBAD*_, and P_*petE*_ (left) and spectrophotometer OD_730_ (right) data sets. The coefficient of variation was calculated for either all the replicates within a laboratory (intralab, in green) or for all the replicates across all laboratories (interlab, orange). In each panel, a boxplot summarizes all data points by showing the median as a horizontal line, the 25th and 75th percentiles as the bottom and top of the box, respectively, and whiskers extending 1.5 × IQR from the box margins. In addition, all data points are shown and distributed over the area of a violin plot.

The measurements performed by all participating laboratories, spectrophotometer OD_730_ and the normalized relative fluorescence units (nRFU) were used as proxies for biomass concentration and mVENUS expression, respectively (see the section Fluorescence Analysis and Normalization in Methods for a detailed explanation of our normalization strategy). We estimated the coefficient of variation (CV) to assess the reproducibility of our protocols by calculating the ratio of the standard deviation to the mean for each time point, strain, and induction regime in the first seven hours of the assay, either for each participant (intralab) or over all locations (interlab) (Figure 1). The CV, inversely proportional to the precision of replicate measurements, was lower at both the intra- and interlab level for the spectrophotometer OD_730_. Measurements from this data set presented a median CV of 6.1% and 11.5% (Figure 1), with 95.4% and 100% of measurements with a CV lower than 20% for intra- and interlab, respectively (Figure S2). On the other hand, the nRFU showed a median CV of 23.9% and 60.6% at the intra- and interlab levels, respectively (Figure 1). In this data set, 43% of intralab replicates had a CV lower than 20%, but at the interlab level, all replicates showed a CV higher than 20% (Figure S2). Even though the nRFU values were less reproducible than the spectrophotometer measurements, this normalization strategy clearly improved the comparability of results across laboratories. This can be seen from the much higher interlab CV values calculated from not normalized values as the background-corrected fluorescence units (FU_bc_) or the relative fluorescence units (RFU) (Figure S3). Interestingly, including the OD_730_ in the normalization procedure did not lead to lower CV at the interlab level (Figure S3). This analysis shows that the measurements performed in the spectrophotometer were the most reproducible in our protocol, which can be partially explained by the fact that the starting conditions of the assay were based on measurements from these devices.

### Discrepancies in Growth Rates Are Not Explained by Different Initial Biomass Concentrations

**Figure 2:**
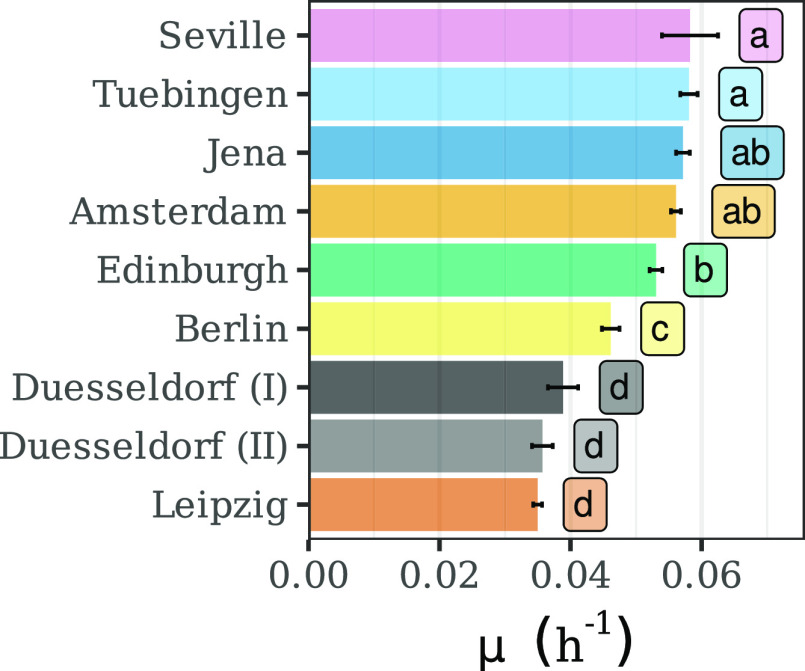
Measured growth rates in each laboratory. Bars represent the mean growth rate and error bars 95% CI (*n* = 32 or 24). Text boxes on the right side of the bars show the results from Tukey’s test. Laboratories with significantly different growth rates (*P*-value < 0.05) are labeled with different letters.

Even though the spectrophotometer results showed the highest reproducibility, we further evaluated if this resulted from highly reproducible culture conditions. To assess this, we used the spectrophotometer OD_730_ to estimate growth rates for each biological replicate over 24 h (Figure 2). This metric is not dependent on the actual OD_730_ values per se but rather on their relationship. The growth rate is known to be influenced by environmental conditions. We performed ANOVA to test if significant differences were found at the strain, induction regime, or laboratory level. The latter was the only factor showing a significant influence (*P*-value < 0.0001) on the measured growth rates.

Therefore, we compared the average growth rate between laboratories using a post hoc Tukey’s test. The results of this analysis showed significant differences (*P*-value < 0.05) between most of the participants. However, the most striking and biologically relevant differences were found between the laboratories, with the highest (Seville, Tuebingen, Jena, and Amsterdam) and lowest growth rates (Duesseldorf and Leipzig). In the first group, growth rates varied between 0.056 and 0.058 h^–1^, while in the second, it ranged between 0.035 and 0.039 h^–1^. This translates into a 36% reduction in the average growth rate.

Our protocols precisely defined the growth conditions (temperature, shaking speed, light intensity) and media composition. However, since we used the spectrophotometer OD_730_ as a reference value to set the initial biomass concentration in the assay, the starting amount of photons per cell could have differed among laboratories. For example, this could have been the case if spectrophotometers used across laboratories had different relationships between OD_730_ and the number of cells.

To test this hypothesis, we performed additional measurements in each spectrophotometer by preparing a dilution of the four glycerol stocks and directly measuring it without allowing cells to grow. The results (Figure S4A) showed that, indeed, there were significant differences (ANOVA, *P*-value < 0.05) across spectrophotometers when measuring the same amount of cells. With this information, we could determine if the differences in growth rates we observed during the assays resulted from the initial biomass concentration. Therefore, we normalized the initial OD_730_ values of the assays by the measurements obtained from the glycerol stocks (Figure S4B). Next, we tested whether growth rates correlated with this relative OD_730_ but found no significant correlation (Pearson’s coefficient: 0.089, *P*-value: 0.834, Figure S4C). Thus, we can conclude that even if the starting amount of photons per cell was not the same across laboratories, this could not explain the observed differences in growth rates.

### P_*petE*_ Expression Across Laboratories Is Less Reproducible than P_*rhaBAD*_ When No Inducer Is Added

**Figure 3:**
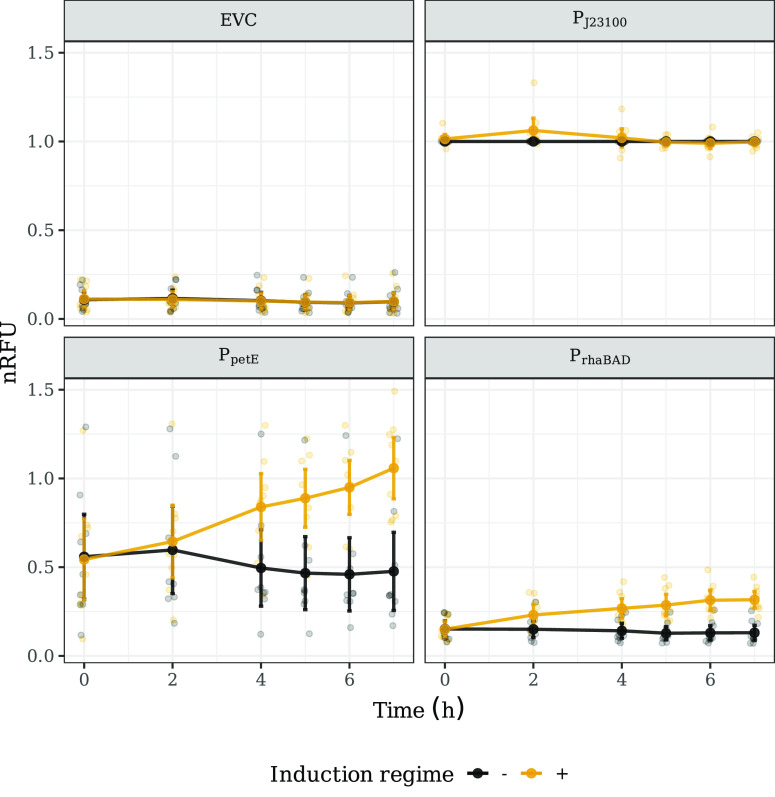
Time series of promoter assay. The nRFU over time is shown for the four strains, each depicted in an individual panel. Cultures, where an inducer was added, are shown in yellow, and those without are shown in black. Smaller data points represent the average values for a single laboratory (*n* = 4 or 3). Larger data points show the overall average and error bars of the overall 95% CI (*n* = 7). The induced and uninduced conditions for EVC and P_*J*23100_ refer to specific aspects of the assay preparation. See the Promoter Quantification Assay section in Methods for further details.

The uninduced P_*J*23100_ (P_*J*23100–_, see the Promoter Quantification Assay section) RFU was used as an internal standard to compare the expression of mVENUS across laboratories (Figure 3). As expected, we observed an average increase in fluorescence during the first seven hours after induction in P_*petE*_ and P_*rhaBAD*_ strains. However, these promoters’ expression patterns differed in reproducibility across laboratories, leakiness, or magnitude of induction.

**Figure 4:**
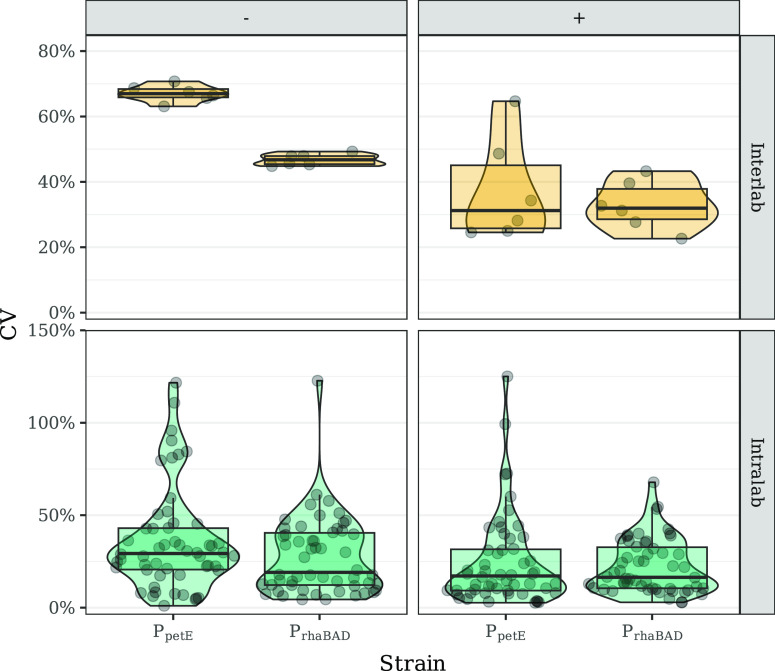
Coefficient of variation (%) of the nRFU measured for all time points in P_*petE*_ and P_*rhaBAD*_ cultures without induction (−, left column) and with induction (+, right column) at the interlab (top row) and intralab level (bottom row). The coefficient of variation was calculated for either all the replicates within a laboratory (intralab, in green) or for all the replicates across all laboratories (interlab, orange). In each panel, a boxplot summarizes all data points by showing the median as a horizontal line, the 25th and 75th percentiles as the bottom and top of the box, respectively, and whiskers extending 1.5 × IQR from the box margins. In addition, all data points are shown and distributed over the area of a violin plot. Notice that the *Y*-axis range is different in the top and bottom rows.

The reproducibility of the nRFU within each laboratory was comparable for both strains and induction regimes (Figure 4). The median CV of the cultures without induction was 19.3% and 29.3% for P_*rhaBAD*_ and P_*petE*_, respectively. Interestingly, the reproducibility across laboratories was higher when an inducer was added (median CV of 16.4% and 17.1% for P_*rhaBAD*_ and P_*petE*_, respectively).

Since we used the P_*J*23100–_ RFU to normalize the RFU values of the other strains, it was impossible to use the nRFU CV to compare the reproducibility of fluorescence measurements across all strains. Thus, we additionally calculated the CV of the not normalized RFU at the intralab level (Figure S5). P_*J*23100_ measurements were the most reproducible among all strains and induction regimes. When no inducer was added, EVC, P_*petE*_, and P_*rhaBAD*_ showed similar reproducibility. However, when the inducer was added, the P_*petE*_ and P_*rhaBAD*_ measurements were more reproducible than the EVC RFU.

As expected, the observed variation of nRFU at the interlab level was higher than within each laboratory (Figure 4). When cultures were induced, increased reproducibility was observed compared to uninduced. A pattern that was already observed on the intralab level comparison. However, while CV values were comparable between both induced strains (median CV of 32% and 31.2% for P_*rhaBAD*_ and P_*petE*_, respectively), the difference between induced and uninduced was much more pronounced. Further, the interlab variability observed in the uninduced P_*petE*_ cultures was much higher than for the uninduced P_*rhaBAD*_ cultures. We observed nonoverlapping distribution of CV values between the two strains, where P_*petE*_’s median CV value was 67% compared to the 46.8% P_*rhaBAD*_’s median CV.

**Figure 5:**
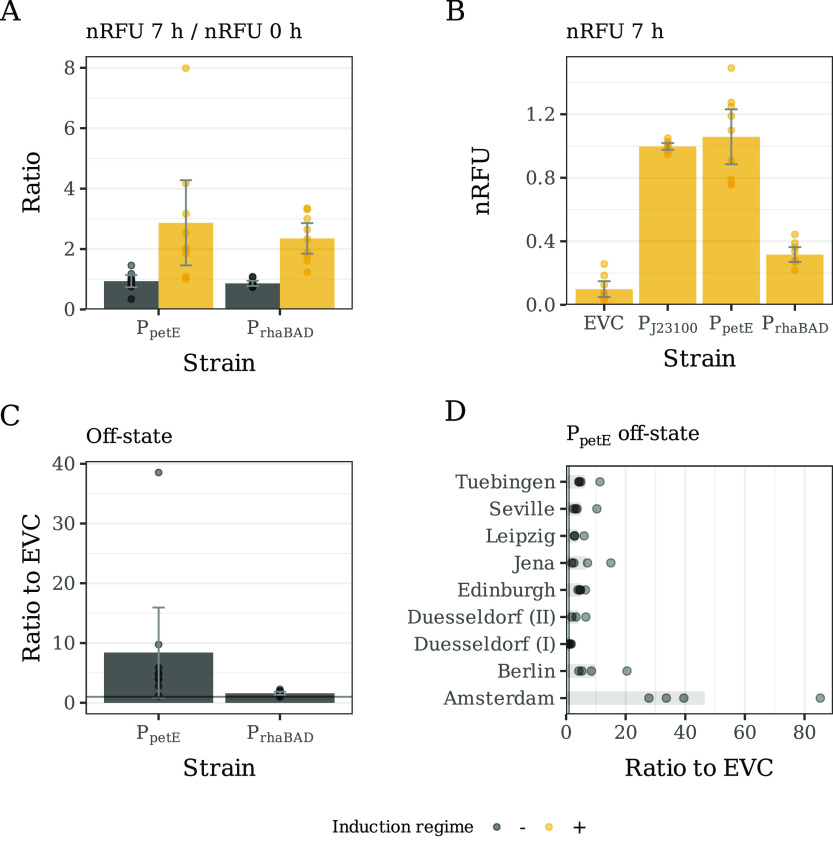
Characterization of P_*petE*_ and P_*rhaBAD*_ promoters. (A) nRFU ratio of time 7 h over time 0 h. (B) nRFU values at time 7 h of induced cultures. (C) nRFU ratio of uninduced PpetE and PrhaBAD to EVC culture at 7 h. (D) nRFU ratio of uninduced PpetE to EVC culture at 7 h in each laboratory. From A to C, points represent the mean value per laboratory (*n* = 3 or 4), bars show the average across all laboratories, and error bars show 95% CI (*n* = 7). In D, points show the mean value of technical replicates from a single experiment (*n* = 3), and bars show the average from each laboratory (*n* = 3 or 4). The color of bars and points indicate the induction regime, black for uninduced and yellow for induced cultures. In C and D, the horizontal and vertical black lines, respectively, represent a ratio of 1.

Inducible promoters can be characterized by measuring a change in expression strength between induced “ON” and uninduced “OFF” states. We, therefore, investigated how these parameters varied between the P_*rhaBAD*_ and P_*petE*_ strains and how they varied between laboratories. We calculated the changes in transcription activity upon induction by dividing the nRFU at seven hours by the value at the beginning of the assay for both induced and uninduced cultures (Figure 5A). When the inducer was absent, the transcriptional activity did not change over the experiment as expected. Changes for all strains in the absence of an inducer were close to 1, with a mean change of 0.86, 95% CI [0.77, 0.95] for P_*rhaBAD*_ and 0.93, 95% CI [0.73, 1.13] for P_*petE*_. When an inducer was added, P_*petE*_ cultures showed the largest average change (2.87, 95% CI [1.46, 4.28]) while also showing high variability between laboratories (Figure 5A). On the other hand, P_*rhaBAD*_ cultures showed an average change of 2.35, 95% CI [1.84, 3.76] with less variability across laboratories.

Regarding expression strength, we observed that after seven hours of induction and with the addition of 1 μM of CuSO_4_, the P_*petE*_ promoter led to similar mVENUS expression levels as the P_*J*23100_ promoter (average 106% of P_*J*23100_, 95% CI [89%, 123%]) (Figure 5B). Under the same conditions and with 10 mM rhamnose, the average expression of the P_*rhaBAD*_ was 32% of P_*J*23100_ (95% CI [27%, 37%]).

The leakiness of an inducible promoter refers to its basal expression level in the absence of the inducer. To assess the P_*petE*_ and P_*rhaBAD*_ promoters’ leakiness, we calculated the ratio of RFU OD_730_^–1^ at seven hours in the uninduced cultures to the EVC (Figure 5C). This ratio is equal to 1 if no signal from the fluorescent reporter is measured in the uninduced condition and will increase proportionally to the leakiness of the promoter. The uninduced P_*rhaBAD*_ cultures showed an average ratio of 1.6, 95% CI [1.3, 1.9]. Conversely, the mean ratio in the P_*petE*_ cultures was 8.4, 95% CI [0.9, 15.9] when no inducer was added, suggesting that either the promoter was very leaky or the environment contained too many residual copper ions (Figure 5C). The high variability observed across the different laboratories (Figure 5D), with an average ratio within laboratories ranging from 1.2 to 38.5 and a median value of 4.8, suggests that residual copper present in the medium of some of the participating laboratories is responsible.

Across eight different laboratories, the growth of PCC 6803 was quantified via OD_730_ measurements, and the transcriptional activity of three promoters (P_*J*23100_, P_*rhaBAD*_, and P_*petE*_) was quantified using fluorescence intensity from mVENUS as a proxy. Reproducibility was evaluated by using inter- and intralaboratory coefficients of variation. We discovered that OD_730_ values showed high reproducibility, albeit the absolute OD_730_ values were not normalized between different laboratories. The fluorescence intensity values of reporters were less reproducible than OD_730_. Generally, fluorescence intensity values of induced promoters were more reproducible than uninduced promoters.

### Reproducibility Is Not Homogeneous Across the Different Measurement Methods

The spectrophotometer measurements in the growth assay showed the highest reproducibility (Figure 1). However, this was expected since the initial OD_730_ of the assay was determined from this device. Nevertheless, the growth rates estimated from these measurements showed significant differences across the participating laboratories (Figure 2). It is well-known that OD measurements are only comparable across devices with additional calibrations.^17^ However, as this study is intended to investigate experimental variation according to commonly used experimental practices, we were interested in the effect of this variation on the experimental results. Thus, we chose not to calibrate the starting OD_730_ by cell counts across laboratories. Instead, calibration measurements were initially performed to later infer the influence of differences in OD_730_ measurements on the overall reproducibility of the study. In these calibration measurements, the reported OD_730_ varied more than 5-fold between participants, confirming the high variability of bulk OD_730_ measurements between spectrophotometer devices. Although this shows that internally, OD_730_ measurements were very reproducible (Figure 1), they were practically incomparable across laboratories without normalization. Therefore, the starting OD_730_ target at 0 h does not represent the same biomass concentration across different laboratories.

Initially, we expected to find a correlation between the growth rate and the normalized initial biomass concentration. However, our data did not reveal such a correlation (Figure S4C), and it remains a matter of speculation which factor caused the differences in the growth rates. It is possible that these differences arose from variations in the light spectra since these are known to affect this physiological parameter.^26,27^ In our protocol, the light color was specified as “white”, which is how it is commonly defined in scientific literature. White light, however, is a combination of different wavelengths, and light spectra across laboratories could have been different. Thus, we compared the light spectra across laboratories and incubators. However, most of the laboratories did not have the instruments to measure the light spectra accurately; thus, we relied, in most cases, on manufacturer specifications. Unfortunately, those specifications were, at best, if at all available, a picture of the light spectrum. After aligning all available spectra and comparing the peaks, we did not find any conclusive trend that explains the observed differences in growth rate.

The plate-reader measurements resulted in less reproducible data at the intra- and interlab level than the spectrophotometer measurements (Figure 1). This observation can be partially explained by the fact that the initial conditions of the assay were set based on the latter device. In addition, several other factors could have influenced the reproducibility of the estimated RFU. First, two different measurements were performed to obtain these values and, thus, two different sensors (OD and fluorescence), increasing the chances of measurement errors. Next, it has been shown that fluorescence measurements of weak promoters (such as P_*rhaBAD*_) are less reproducible due to the poor sensitivity of plate readers at low signals.^21,22^ Lastly, the inclusion of two different induction regimes, which certainly affect the fluorescent output but not the growth behavior, might have contributed to the lower reproducibility of the estimated RFU × OD_730_^–1^ compared to the spectrophotometer measurements.

### Uninduced Reporter Systems Show a Higher Level of Variation Compared to Systems Induced above Saturation Levels

Interestingly, the promoter activity of inducible promoters was more reproducible in the presence than in the absence of the respective inducer. Introducing an additional experimental step (addition of the inducer) did not lead to a higher level of variation. Further, the observed significant differences between measured growth rates did not seem to translate into high variation in promoter activity, highlighting the robustness of those promoters across different physiological stages of cells. Lastly, the differences in initial cell biomass, inferred by differences in OD_730_ values of the identical stock between laboratories, resulted in differences in the inducer/cell count ratio. However, as induction was performed above saturation concentration for the inducer based on previous experiments,^6^ the investigated promoters both seem to perform relatively robustly toward variations in inducer/cell count ratio.

It is important to note that the expression level of the P_*rhaBAD*_ promoter in the OFF state is extremely low and barely above background signals. Thus, much of the intralab CV of the P_*rhaBAD*_ can be attributed to the background noise and not variations in expression level (compare Supplemental Figure S5). The P_*petE*_ system shows higher location-dependent variation. Whereas residual copper could explain the higher variance in the P_*petE*_ promoter system, this can be excluded for P_*rhaBAD*_ as rhamnose is unlikely to be naturally present in trace concentrations. Varying copper residuals on glassware or in filtered water are expected to be the main culprit of this issue. Copper is a ubiquitously present metal ion that is difficult to remove altogether. Acid-washing glassware prior to experiments^28^ could help here. Additionally, the use of chelators such as bathocuproinedisulfonic acid disodium salt^14^ in the medium is a way to reduce copper availability efficiently during the experiment. For future studies employing the P_*petE*_ promoter in real applications, we recommend supporting the data with a fluorescence activity-based assay, as done in this study. This additional experiment should be done in the same strain used in the application and under similar experimental conditions. This process control not only helps to approximate the activity of the P_*petE*_ promoter in the actual experiment but also serves as a process control that can be used by investigators trying to replicate the results. As our results not only show high activity and variance in the OFF state of the P_*petE*_ promoter, we recommend carefully observing experimental results of a strain with a seemingly uninduced P_*petE*_ promoter. Furthermore, these native problems of the P_*petE*_ promoter might be mitigated in the future by further engineering the promoter system: heterologous coexpression of the petR/petP regulators may positively impact the sensitivity to residual copper.

### Strategies to Improve Comparability and Reproducibility

With the increasing throughput of experiments, improving reporting and reproducibility standards becomes increasingly important. None of the following recommendations are definite answers to this question. Instead, they are intended to provoke discussions and reflections about the reproducibility of cyanobacterial research.

A good strain-handling policy is important for keeping research results reproducible over time. Especially for bacterial strains, this is essential since genetic changes occur rapidly. Those changes resulting from improper strain maintenance lead to genotypic diversity and consequently to conflicts in research findings. The effect of the genetic diversity of PCC 6803 and its impact on phenotype has been analyzed in the past.^29,30^ Cryopreservation techniques have been established for long-term microbial evolution experiments to reduce genotypic variability between experiments with the same strain.^31^ To our knowledge, such techniques are not standard practice in the cyanobacterial field. In this study, we aimed to minimize strain variability not only between participants but also between experimental runs within a single location. Therefore, we adapted a cryopreservation and inoculation protocol based on glycerol conservation from Price et al.^31^ to the PCC 6803 strains used in this interlab study. With this protocol, we hope to contribute to standardizing strain handling for cyanobacteria, especially for PCC 6803.

Next to varying practices in strain-keeping, the preparation of BG11 medium is a source for variations. During the establishment of the experimental procedures, we encountered that the preparation and final mass concentration of almost all solutes in BG11 media differed across participating laboratories. In addition, we obtained similar outcomes when comparing BG11 media protocols published in the scientific literature. Regarding standardization, we agreed on the formula developed by van Alphen et al.,^32^ except for removing copper from the media for our particular purposes. Additionally, as outlined in the methods, a weak HEPES buffer was added to standardize starting conditions after inoculation. As cyanobacterial cultures rapidly increase the pH of their environment during cultivation, this buffer is assumed to be nonsignificant in prolonged cultivation. However, we assumed it would be better to buffer initial pH disruptions during inoculation. It remains open if those changes in BG11 media would substantially affect the experiments. However, we would encourage everyone also to standardize the preparation of BG11 as most differences in the preparation have no fundamental reason other than long-lasting traditions in respective laboratories.

In addition, when working with phototrophs, reporting the exact specification of light spectra used would be an important addition to reporting growth conditions. However, measuring light spectra requires a light meter, which is not standard laboratory equipment. Furthermore, manufacturers of light bulbs do not disclose those specifications in most cases or in an inappropriate format. Thus, we encourage manufacturers of light bulbs to report the light spectra as a CSV file with raw values. Further, we would encourage scientists to ask for the light spectra when buying a new incubator or contact the manufacturer to request the light spectra of your current incubators.

The iGEM interlab studies have gathered a considerable amount of data and protocols that support the use of external calibrants for both optical density and fluorescence for promoter characterization.^20−23^ There are indeed clear benefits in defining such protocols. By calibrating plate readers (or flow cytometers) with external standards for fluorescence and cell concentration, promoter activity can be reported in absolute units, allowing direct comparisons across laboratories and facilitating the detection of biological and technical errors.^20−23^ However, these protocols are not universally applicable. Regarding fluorescence reporters, GFP is not the only fluorescent protein used in cyanobacterial research, and YFP derivatives such as mVENUS have become popular in recent years.^33^ Therefore, this requires that new calibrants are found for each new fluorescent protein, matching its excitation and emission wavelengths.

It has been known that OD measurements differ between instruments and, thus, laboratories.^17,34^ Hence, only reporting OD values as a measure for cell biomass is problematic but still common practice in microbiology. The Stevenson et al. and later the iGEM interlab studies proposed using materials that match the refractive index of *E. coli* to calibrate OD.^20−23,34^ In addition to being species-specific, this approach presents the disadvantage of assuming that cells of a given species are always the same size and shape. An option to overcome this problem would be to report cell counts and size with the starting OD values so that individual researchers can calibrate their OD measurements to the respective cell count.^35^ We are aware that cell counters are not always available in all laboratories, as was the case among the participants of this study. However, we encourage reporting cell counts in combination with OD for better reproducibility in the future.

Our alternative to overcome these issues was using an internal biological standard. In our experimental design, we employed P_*J*23100_ as the normalizing promoter within each location, therefore accounting for differences between instruments. In addition, this approach also excludes factors such as the intracellular abundance of RNA polymerases and sigma factors, ribosomes, and other components of protein production impacting the mVENUS expression and maturation. Increased mVENUS expression from one of the inducible promoters related to any of these factors was expected to affect the mVENUS expression in the J23100 strain equally. As all our RFU values are reported as RFU_strain_/RFU_*J*23100_, these factors are presumably either buffered or completely negated. Considering that no significant differences in growth rates between the strains were found within each laboratory and identical absorption spectra across all the used strains in all laboratories, highly similar growth conditions can be assumed for all data sets. By correcting these factors, this normalization approach enables enhanced comparability across laboratories. This is demonstrated by the reduced interlab CV in nRFU, compared to the non-normalized metrics (Figure S3). It is worth noting that we observed a slightly lower coefficient of variation (CV) between laboratories when employing FU_bc_ instead of RFU for our normalization method (Figure S3). Essentially, including OD_730_ in the procedure led to an increased variation due to the intrinsic error of these measurements propagating into nRFU. Although this small increase in variation is present, we consider it justified as it addresses potential variation in fluorescence due to cultures with different optical densities. Therefore, we recommend such a normalization strategy to report more reproducible data.

Lastly, our experimental setup used the native copper repressible promoter P_*petE*_ and the heterologous P_*rhaBAD*_ system as a somewhat orthologous regulation system. Even though the BG11 medium used in this study was prepared without adding copper salts, copper is hard to remove from glassware, and even a slight amount of residual copper could have caused the differences in basal activity observed in our experiments. Additionally, as a native promoter, P_*petE*_ may be subjected to more levels of regulation than so far known, which could lead to greater variability across different laboratories. Thus, we encourage the use of orthogonal systems like the P_*rhaBAD*_ promoter, which overall performed better in this study in terms of reproducibility.

Considering the orthogonal nature of the P_*rhaBAD*_ promoter in PCC 6803 and assuming no residual rhamnose in uninduced media, we hypothesize that the interlaboratory reproducibility of this experimental procedure and the multitude of measures taken to ensure maximum comparability of results across this study, we propose that the coefficients of variation of promoter activity for this system could indicate where the baseline for maximum reproducibility of quantitative data in PCC 6803 lies. Essentially, the coefficient of variation for the P_*rhaBAD*_ promoter serves as a call for caution when it comes to the overall reproducibility of fluorescence activity—and potentially other—data in the field of cyanobacterial research and a call for action to aid in improving comparability by following the discussed strategies: standardized and rigid strain keeping policies, standardized media composition, thorough reporting of cultivation conditions and the correct use of normalization agents.

## Conclusion

In conclusion, the reproducibility of promoter activity in cyanobacteria within this study across multiple laboratories was better than expected. However, we also identified some causes for errors that could be improved in future studies. The remaining problem when using cyanobacteria is that most options to correct for those errors are understudied or completely missing, and those tools would first need further development to increase reproducibility.

## Methods

The strains for this study were generated in a single laboratory (Duesseldorf) and distributed to all participants. The interlab experiment was performed following a detailed set of protocols, which can be found at 10.17504/protocols.io.3byl4j69rlo5/v1. See Figure S1 for a visual description of the protocol.

### Plasmid and Strain Construction

The plasmids were constructed via Golden Gate Cloning.^1^ Complete and annotated sequences of all plasmids are available in supplementary data on Figshare (10.6084/m9.figshare.21525747.v5). According to standard procedures, *Escherichia coli* DH5α cells were transformed with Golden Gate mixes according to the MoCloFlex protocol.^36^ Sequences were verified via Sanger sequencing. PCC 6803 was conjugated via triparental mating,^37^ and conjugants were confirmed via colony PCR.

The PCC 6803 strain (glucose tolerant, nonmotile) was obtained from D. Bhaya (Carnegie Institution for Science, Stanford, USA).

### Media

All participants prepared a BG11 medium without CuSO_4_ supplemented with 10 mM NaHCO_3_ and 5 mM HEPES-NaOH (pH 8). The exact composition of BG11 and the detailed recipe for the stock’s preparation can be found in the supplements (Table S3 and S4) and was extracted from the Supporting Data S3 document of van Alphen et al.^32^ It is hereafter referred to as BG11. For each repetition of the assay, 1 L of BG11 was prepared fresh for each round of experiments: First, approximately 500 mL of ultrapure H_2_O were autoclaved in a 1 L volumetric bottle. Then stock solutions were acclimated to room temperature and added in the following order: 5 mL of 1 M HEPES-NaOH, 2.5 mL BG11 S1, 2.5 mL BG11 S2 (without CuSO_4_), 2.5 mL BG11 S3, and 10.5 mL 0.95 M NaHCO_3_. After adding the solutions, sterile ultrapure H_2_O was added up to the 1 L mark of the bottle.

### Cultivation for Strain Conservation

For the preparation of cryoconserved cultures, PCC 6803 was grown under the same conditions outlined in Culture Conditions in Interlab Experiment, except for increasing the light intensity to 80 μ mol photons × m^–2^ × s^–1^. When the OD measured at 730 nm wavelength (hereafter OD_730_) reached a value of 3, cells were centrifuged at 12,000*g* (fixed angle rotor) for 15 min, washed with fresh BG11 medium, centrifuged once again, and resuspended in 10% of the initial volume with BG11 and 15% (v/v) final concentration glycerol. Cells were stored at −80 °C and shipped to participating laboratories on dry ice. The incubator and photometer listed in Table S5 under “Duesseldorf I” were used to prepare cryoconserved cultures.

### Participants

Eight different laboratories participated in the study and are described throughout the study by the geographical location of the research facility. In alphabetical order, the participants were from Amsterdam (University of Amsterdam), Berlin (Freie Universität Berlin), Duesseldorf (Heinrich Heine University), Edinburgh (University of Edinburgh), Jena (Friedrich Schiller University), Leipzig (Helmholtz Centre for Environmental Research), Seville (University of Seville), and Tuebingen (University of Tübingen), as included in the author list. In Duesseldorf, the complete experiment was independently performed by two researchers, indicated in the text as Duesseldorf (I) and Duesseldorf (II). Strains were prepared and grown in Duesseldorf and shipped to participants on dry ice.

### Culture Conditions in Interlab Experiment

Cultures were grown in 100 mL Erlenmeyer nonbaffled glass flasks with cotton plugs in shaking incubators set at 100 rpm (see Table S5) under 50 μmol photons × m^–2^ × s^–1^ constant white light illumination, ambient CO_2_, and 30 °C (except for Tuebingen where the temperature was 28 °C). Cultures were grown in BG11 medium, as outlined above. Cultures were always grown with 10 μg mL^–1^ chloramphenicol, which was added individually to each flask before inoculation.

### Promoter Quantification Assay

PCC 6803 strains harboring plasmids containing the investigated promoter-reporter cassette were inoculated from cryoconserved cultures by adding 330 μL of inoculum to 10 mL of copper-free BG11 medium supplied with chloramphenicol and cultivated for 48 h. Cultures were then diluted to an OD_730_ of 0.3 in 35 mL and grown overnight to OD_730_ of 0.5–0.6. The following day, the complete volume of each preculture was transferred to a sterile tube and diluted to OD_730_ 0.5 if necessary. The flasks where the precultures had been grown were washed twice with copper-free BG11. Subsequently, cultures were separated into two times 20 mL of OD_730_ 0.5 in two separate sets of flasks, which were labeled as induced and uninduced. The washed flasks were used for the uninduced cultures and were supplied with 200 μL of ultrapure H_2_O. In the induced set, 200 μL of 200 μM CuSO_4_ or 1 M rhamnose, were supplied to the P_*petE*_ and P_*rhaBAD*_ flasks, respectively. In the EVC and P_*J*23100_ cultures, 200 μL of ultrapure H_2_O was supplied. Throughout the text, we refer to induced and uninduced cultures of EVC and P_*J*23100_, although they do not carry an inducible promoter, and ultrapure H_2_O was supplied in all cases. Therefore for these strains, this nomenclature should be interpreted as cultures that were grown in the washed flasks (uninduced) and those that grew in the new flasks (induced). Reusing the preculture flask was an attempt to remove residual copper from the flask where the uninduced P_*petE*_ culture would be grown. The rationale behind it was that by growing a culture in a free-copper medium, cells could potentially take up residual copper from the glassware. To maintain a homogeneous protocol for all strains, this step was also performed on the other three strains.

### Sampling and Measurements

In total, seven samples were taken from each flask. Samples were taken at 0, 2, 4, 5, 6, 7, and 24 h after adding the inducers in the promoter quantification assay. In most cases, 1 mL of culture was taken at each sampling point except for those laboratories that performed chlorophyll extraction at times 0, 7, and 24 h, in which case the sample volume was 2 mL.

As mentioned above, OD_730_ was measured in a spectrophotometer at a 730 nm wavelength (see Table S5 for models at each laboratory). For spectrophotometer measurements, samples were diluted 1:2 (500 μL:500 μL) for the same-day measurements and 1:5 (200 μL:800 μL) for the 24-h measurements. For plate reader measurements, 100 μL of liquid cultures were used without further dilution for same-day measurements and 100 μL of 1:5 (20 μL:80 μL) dilution for 24 h measurements.

The mVENUS fluorescence intensity was measured in a plate reader (see Table S5 for models at each laboratory) using an excitation window of 506–518 nm, and emission was detected in a 542–562 nm window.

The 24 h time point was excluded from the data analysis for technical reasons. To ensure the comparability of measurements within each laboratory, we used the same plate reader settings for all measurements. While this worked well for all time points, including the 7 h measurement, culture cell densities at the 24 h time point were beyond the linear sensitivity range. Simultaneously the dilution of the culture at 24 h caused some measurements to be at the lower plate reader sensitivity level for the low-density cultures. In both cases, measurements produced unreliable results that made data analysis impossible. We encountered this issue within the running study and did not have enough identical starting cultures in each laboratory to revise the study’s design. This issue highlights the problems of reproducing experiments in other laboratories with different equipment if no further precautions and tests are implemented before doing the actual study. Thus, reproducing other results requires substantial modifications to the setup of instruments and workflow across laboratories in some circumstances. However, for the purpose of the study, the included data points are sufficient to analyze the reproducibility of the promoters used in this study.

### Chlorophyll Extraction

One milliliter of cyanobacterial culture was centrifuged at 10,000*g* for 5 min. 900 μL of supernatant was discarded, and the pellet was resuspended in the remaining 100 μL. Next, 900 μL of 100% methanol was added and mixed thoroughly by vortexing. Samples were incubated in the dark at 4 °C for 5 min and centrifuged again at 10,000*g* for 5 min. The supernatant was transferred to a cuvette, and extinction was measured at 665 nm using a spectrophotometer. 900 μL methanol mixed with 100 μL BG11 was used as the reference solution. To estimate chlorophyll concentration from the absorbance at 665 nm, eq 1 was used. The extraction protocol and eq 1 were adapted from Ritchie.^38^
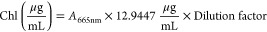
1

### Data Collection

The assay was performed four times independently in each laboratory. The four strains used in each experimental run were inoculated from an individual glycerol stock and, therefore, considered a biological replicate from each strain, induction regime, and experimental run. Each biological replicate included seven measurement points at 0, 2, 4, 5, 6, 7, and 24 h after inoculation. In the case of the plate reader, each biological replicate was measured in three independent wells at each time point.

### Data Analysis

Data from all participants were submitted in a standardized spreadsheet file containing all measurements from a single experimental run. All subsequent analyses to process the data and create the figures in this manuscript were carried out in R.^39^ The following R packages were used in the analysis: agricolae,^40^ broom,^41^ ggforce,^42^ ggthemes,^43^ janitor,^44^ patchwork,^45^ readxl,^46^ and tidyverse.^47^ The data, as well as the code of the complete analysis and figures, are available at https://github.com/hugo-pH/cyano_interlab. The raw data are also available at 10.6084/m9.figshare.21525747.v5.

### Growth Rates

Growth rates were estimated from the spectrophotometer OD_730_ data by linear regression, using the log-transformed equation of exponential growth (ln OD_730_*t* = μ × *t* + ln OD_730_*t*_0_), where μ denotes the growth rate. All time points were used in this analysis.

### Fluorescence Analysis and Normalization

Both raw OD_730_ and fluorescence units (FU) from the plate reader data set were first background-corrected by subtracting the average value of the blank wells at each time point (eqs 2 and 3). In addition, the smallest FU value measured in each experimental run and location was added to all data points to avoid negative FU values. Next, relative fluorescence units (RFU) were calculated by dividing the background-corrected FU by the background-corrected OD_730_ for each technical replicate (eq 4), followed by averaging all technical replicates. Since the scale of OD_730_ and FU recorded by plate readers greatly varies between devices, a normalization method was implemented to compare results across different devices. Instead of using an external calibrant, the RFU of P_*J*23100_ cultures without an inducer was used as an internal biological standard for plate reader measurements. This strain was chosen because it expresses mVENUS constitutively. This approach can correct not only the different measuring ranges of plate reader devices but also possible differences in the physiological state due to discrepancies in growth conditions across the experimental runs (and across laboratories). Finally, the RFU of each biological replicate and time point was divided by the corresponding P_*J*23100_ RFU value (eq 5) to apply this method, obtaining the normalized RFU (nRFU).
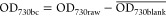
2
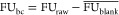
3
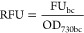
4
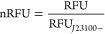
5

## Supporting Information Available

Additional analysis of variations of plate reader and spectrophotometer measurements, an equipment list for each participating laboratory, and a detailed description of the medium preparation (PDF)

## Abbreviations

nRFU: normalized relative fluorescence units
BCD2: bicistronic design.

## References

[ref1] Weber E.; Engler C.; Gruetzner R.; Werner S.; Marillonnet S. A Modular Cloning System for Standardized Assembly of Multigene Constructs. PLoS One 2011, 6, 1–11. 10.1371/journal.pone.0016765. PMC3041749 21364738

[ref2] Ungerer J.; Pakrasi H. B. Cpf1 Is A Versatile Tool for CRISPR Genome Editing Across Diverse Species of Cyanobacteria. Sci. Rep. 2016, 10.1038/srep39681. PMC5175191 28000776

[ref3] Liu D.; Johnson V. M.; Pakrasi H. B. A Reversibly Induced CRISPRi System Targeting Photosystem II in the Cyanobacterium *Synechocystis* sp. PCC 6803. ACS Synth. Biol. 2020, 9, 1441–1449. 10.1021/acssynbio.0c00106. 32379958

[ref4] Sode K.; Tatara M.; Takeyama H.; Burgess J. G.; Matsunaga T. Conjugative gene transfer in marine cyanobacteria: *Synechococcus* sp., *Synechocystis* sp. and *Pseudanabaena* sp. Appl. Microbiol. Biotechnol. 1992, 37, 369 10.1007/BF00210994. 1368911

[ref5] Vasudevan R.; Gale G. A. R.; Schiavon A. A.; Puzorjov A.; Malin J.; Gillespie M. D.; Vavitsas K.; Zulkower V.; Wang B.; Howe C. J.; Lea-Smith D. J.; McCormick A. J. CyanoGate: A Modular Cloning Suite for Engineering Cyanobacteria Based on the Plant MoClo Syntax. Plant Physiology 2019, 180, 39–55. 10.1104/pp.18.01401. 30819783 PMC6501082

[ref6] Behle A.; Saake P.; Germann A. T.; Dienst D.; Axmann I. M. Comparative Dose–Response Analysis of Inducible Promoters in Cyanobacteria. ACS Synth. Biol. 2020, 9, 843–855. 10.1021/acssynbio.9b00505. 32134640

[ref7] Gale G. A. R.; Wang B.; McCormick A. J. Evaluation and Comparison of the Efficiency of Transcription Terminators in Different Cyanobacterial Species. Front. Microbiol. 2021, 11, 11 10.3389/fmicb.2020.624011. PMC7843447 33519785

[ref8] Englund E.; Pattanaik B.; Ubhayasekera S. J. K.; Stensjö K.; Bergquist J.; Lindberg P. Production of Squalene in *Synechocystis* sp. PCC 6803. PLoS One 2014, 9, 1–8. 10.1371/journal.pone.0090270. PMC3953072 24625633

[ref9] Geerts D.; Bovy A.; de Vrieze G.; Borrias M.; Weisbeek P. Inducible expression of heterologous genes targeted to a chromosomal platform in the cyanobacterium *Synechococcus* sp. PCC 7942. Microbiology 1995, 141, 831–841. 10.1099/13500872-141-4-831. 7773387

[ref10] El-Shehawy R. M.; Kleiner D. Effect of controlled expression of the hetR gene on heterocyst formation in the filamentous cyanobacterium *Anabaena* sp. PCC 7120. Physiologia Plantarum 2003, 119, 44–48. 10.1034/j.1399-3054.2003.00108.x.

[ref11] Ferreira E. A.; Pacheco C. C.; Pinto F.; Pereira J.; Lamosa P.; Oliveira P.; Kirov B.; Jaramillo A.; Tamagnini P. Expanding the toolbox for *Synechocystis* sp. PCC 6803: validation of replicative vectors and characterization of a novel set of promoters. Synth. Biol. 2018, 10.1093/synbio/ysy014. PMC7445879 32995522

[ref12] Giner-Lamia J.; López-Maury L.; Florencio F. J. Global Transcriptional Profiles of the Copper Responses in the Cyanobacterium *Synechocystis* sp. PCC 6803. PLoS One 2014, 9, 1–16. 10.1371/journal.pone.0108912. PMC4182526 25268225

[ref13] Kelly C. L.; Taylor G. M.; Hitchcock A.; Torres-Méndez A.; Heap J. T. A Rhamnose-Inducible System for Precise and Temporal Control of Gene Expression in Cyanobacteria. ACS Synth. Biol. 2018, 7, 1056–1066. 10.1021/acssynbio.7b00435. 29544054

[ref14] García-Cañas R.; Giner-Lamia J.; Florencio F. J.; López-Maury L. A protease-mediated mechanism regulates the cytochrome *c*_6_/plastocyanin switch in *Synechocystis* sp. PCC 6803. Proc. Natl. Acad. Sci. U. S. A. 2021, 118, e2017898118 10.1073/pnas.2017898118. 33495331 PMC7865156

[ref15] Englund E.; Liang F.; Lindberg P. Evaluation of promoters and ribosome binding sites for biotechnological applications in the unicellular cyanobacterium *Synechocystis* sp. PCC 6803. Sci. Rep. 2016, 10.1038/srep36640. PMC5114575 27857166

[ref16] Baker M. 1,500 scientists lift the lid on reproducibility. Nature 2016, 533, 452 10.1038/533452a. 27225100

[ref17] Myers J. A.; Curtis B. S.; Curtis W. R. Improving accuracy of cell and chromophore concentration measurements using optical density. BMC Biophys. 2013, 10.1186/2046-1682-6-4. PMC3663833 24499615

[ref18] Tietze L.; Mangold A.; Hoff M. W.; Lale R. Identification and Cross-Characterisation of Artificial Promoters and 5 Untranslated Regions in *Vibrio natriegens*. Front. Bioeng. Biotechnol. 2022, 10.3389/fbioe.2022.826142. PMC8830501 35155395

[ref19] Interlaboratory Comparisons. EU Science Hub. European Commission, 2022. https://joint-research-centre.ec.europa.eu/reference-measurement/interlaboratory-comparisons_en (Accessed April 19, 2022).

[ref20] Beal J.; Haddock-Angelli T.; Gershater M.; de Mora K.; Lizarazo M.; Hollenhorst J.; Rettberg R. Reproducibility of Fluorescent Expression from Engineered Biological Constructs in *E. coli*. PLoS One 2016, 11, 1–22. 10.1371/journal.pone.0150182. PMC4777433 26937966

[ref21] Beal J.; Haddock-Angelli T.; Baldwin G.; Gershater M.; Dwijayanti A.; Storch M.; de Mora K.; Lizarazo M.; Rettberg R. Quantification of bacterial fluorescence using independent calibrants. PLoS One 2018, 13, 1–15. 10.1371/journal.pone.0199432. PMC6013168 29928012

[ref22] Beal J.; Farny N. G.; Haddock-Angelli T.; Selvarajah V.; Baldwin G. S.; Buckley-Taylor R.; Gershater M.; Kiga D.; Marken J.; Sanchania V.; Sison A.; Workman C. T. Robust estimation of bacterial cell count from optical density. Commun. Biol. 2020, 10.1038/s42003-020-01127-5. PMC7591534 33110148

[ref23] Beal J.; et al. Comparative analysis of three studies measuring fluorescence from engineered bacterial genetic constructs. PLoS One 2021, 16, 1–15. 10.1371/journal.pone.0252263. PMC8183995 34097703

[ref24] Egan S. M.; Schleif R. F. A Regulatory Cascade in the Induction of rhaBAD. J. Mol. Biol. 1993, 234, 87–98. 10.1006/jmbi.1993.1565. 8230210

[ref25] Mutalik V. K.; Guimaraes J. C.; Cambray G.; Lam C.; Christoffersen M. J.; Mai Q.-A.; Tran A. B.; Paull M.; Keasling J. D.; Arkin A. P.; Endy D. Precise and reliable gene expression via standard transcription and translation initiation elements. Nat. Methods 2013, 10, 354–360. 10.1038/nmeth.2404. 23474465

[ref26] Bland E.; Angenent L. T. Pigment-targeted light wavelength and intensity promotes efficient photoautotrophic growth of Cyanobacteria. Bioresour. Technol. 2016, 216, 579–586. 10.1016/j.biortech.2016.05.116. 27285573

[ref27] Luimstra V. M.; Schuurmans J. M.; Verschoor A. M.; Hellingwerf K. J.; Huisman J.; Matthijs H. C. P. Blue light reduces photosynthetic efficiency of cyanobacteria through an imbalance between photosystems I and II. Photosynthesis Research 2018, 138, 177–189. 10.1007/s11120-018-0561-5. 30027501 PMC6208612

[ref28] Elomaa H.; Seisko S.; Junnila T.; Sirviö T.; Wilson B. P.; Aromaa J.; Lundström M. The Effect of the Redox Potential of Aqua Regia and Temperature on the Au, Cu, and Fe Dissolution from WPCBs. Recycling 2017, 2, 14 10.3390/recycling2030014.

[ref29] Trautmann D.; Voß B.; Wilde A.; Al-Babili S.; Hess W. R. Microevolution in Cyanobacteria: Re-sequencing a Motile Substrain of *Synechocystis* sp. PCC 6803. DNA Research 2012, 19, 435–448. 10.1093/dnares/dss024. 23069868 PMC3514855

[ref30] Zavřel T.; Očenášová P.; Červený J. Phenotypic characterization of *Synechocystis* sp. PCC 6803 substrains reveals differences in sensitivity to abiotic stress. PLoS One 2017, 12, 1–21. 10.1371/journal.pone.0189130. PMC5720811 29216280

[ref31] Price C. E. Adaption to glucose limitation is modulated by the pleotropic regulator CcpA, independent of selection pressure strength. BMC Evol. Biol. 2019, 10.1186/s12862-018-1331-x. PMC6327505 30630406

[ref32] van Alphen P.; Abedini Najafabadi H.; Branco dos Santos F.; Hellingwerf K. J. Increasing the Photoautotrophic Growth Rate of *Synechocystis* sp. PCC 6803 by Identifying the Limitations of Its Cultivation. Biotechnol. J. 2018, 13, 1700764 10.1002/biot.201700764. 29577667

[ref33] Yokoo R.; Hood R. D.; Savage D. F. Live-cell imaging of cyanobacteria. Photosynthesis Research 2015, 126, 33–46. 10.1007/s11120-014-0049-x. 25366827

[ref34] Stevenson K.; McVey A. F.; Clark I. B. N.; Swain P. S.; Pilizota T. General calibration of microbial growth in microplate readers. Sci. Rep. 2016, 6, 38828 10.1038/srep38828. 27958314 PMC5153849

[ref35] Hays S. G.; Yan L. L. W.; Silver P. A.; Ducat D. C. Synthetic photosynthetic consortia define interactions leading to robustness and photoproduction. J. Biol. Eng. 2017, 10.1186/s13036-017-0048-5. PMC5259876 28127397

[ref36] Klein C. A.; Emde L.; Kuijpers A.; Sobetzko P. MoCloFlex: A Modular Yet Flexible Cloning System. Front. Bioeng. Biotechnol. 2019, 10.3389/fbioe.2019.00271. PMC6843054 31750294

[ref37] Behle A. Triparental mating of Synechocystis. protocols.io. December 20, 2016. 10.17504/protocols.io.ftpbnmn.

[ref38] Ritchie R. J. Universal chlorophyll equations for estimating chlorophylls a, b, c, and d and total chlorophylls in natural assemblages of photosynthetic organisms using acetone, methanol, or ethanol solvents. Photosynthetica 2008, 46, 115–126. 10.1007/s11099-008-0019-7.

[ref39] R Core Team. R: A Language and Environment for Statistical Computing; R Foundation for Statistical Computing: Vienna, Austria, 2013.

[ref40] de Mendiburu F. agricolae: Statistical Procedures for Agricultural Research; R Package version 1.3.1; 2019.

[ref41] Robinson D.; Hayes A.; Couch S. broom: Convert Statistical Objects into Tidy Tibbles; R package version 0.7.10; 2021.

[ref42] Pedersen T. L. ggforce: Accelerating ggplot2; R package version 0.4.1; 2022.

[ref43] Arnold J. B. ggthemes: Extra Themes, Scales and Geoms for ggplot2; R package version 4.2.4; 2021.

[ref44] Firke S. janitor: Simple Tools for Examining and Cleaning Dirty Data; R package version 2.1.0; 2021.

[ref45] Pedersen T. L. patchwork: The Composer of Plots; R package version 1.1.2; 2022.

[ref46] Wickham H.; Bryan J. readxl: Read Excel Files; R package version 1.3.1; 2019.

[ref47] Wickham H.; et al. Welcome to the tidyverse. Journal of Open Source Software 2019, 4, 1686 10.21105/joss.01686.

