# Supplementary material for: Interlaboratory Reproducibility in Growth and Reporter Expression in the Cyanobacterium Synechocystis sp. PCC 6803
Source: ACS Synth Biol. 2023 May 29;12(6):1823–35. doi: 10.1021/acssynbio.3c00150 (PMC10278186; doi:10.1021/acssynbio.3c00150)
Supplement: Supplementary file 1 — sb3c00150_si_001.pdf [file sb3c00150_si_001.pdf]

Supporting Information

Interlaboratory Reproducibility in Growth and Reporter Expression in the Cyanobacterium Synechocystis sp. PCC 6803

Maurice Mager [†,‡‡] Hugo Pineda Hernandez [‡,‡‡] Fabian Brandenburg, [¶] Luis López-Maury [§,‖] Alistair J. McCormick [⊥] Dennis J. Nürnberg [#,@] Tim Orthwein [△] David A. Russo [∇] Angelo Joshua Victoria [⊥] Xiaoran Wang [#] Julie A. Z. Zedler [††] Filipe Branco dos Santos [*,‡,¶¶] and Nicolas M. Schmelling [*,†,¶¶]

†*Institute for Synthetic Microbiology, Heinrich Heine University Duesseldorf, Universitaetsstrasse 1, 40225 Duesseldorf, Germany*

‡*Molecular Microbial Physiology Group, Swammerdam Institute for Life Sciences, Faculty of Science, University of Amsterdam, Science Park 904, Amsterdam 1098 XH, The Netherlands*

¶*Helmholtz Centre for Environmental Research (UFZ), Permoserstrasse 15, 04318 Leipzig, Germany*

§*Instituto de Bioquímica Vegetal y Fotosíntesis, University of Seville - CSIC, Américo Vespucio 49, 41092 Sevilla, Spain*

‖*Departamento de Bioquímica Vegetal y Biología Molecular, Facultad de Biología, University of Seville, Avenida Reina Mercedes, 41012 Sevilla, Spain*

⊥*Institute of Molecular Plant Sciences, School of Biological Sciences, University of Edinburgh, 1.04 Daniel Rutherford Building, King's Buildings, EH9 3BF, Edinburgh, UK*

#*Freie University Berlin, Department of Physics, Experimental Biophysics, Arnimallee 14, 14195 Berlin, Germany*

@*Dahlem Centre of Plant Sciences, Freie Universität Berlin, Albrecht-Thaer-Weg 6, 14195 Berlin, Germany*

△*University of Tuebingen, Interfaculty Institute of Microbiology and Infection Medicine, Auf der Morgenstelle 28, 72076 Tübingen, Germany*

∇*Friedrich Schiller University Jena, Institute for Inorganic and Analytical Chemistry, Bioorganic Analytics, Lessingstrasse 8, 07743 Jena, Germany*

††*Friedrich Schiller University Jena, Matthias Schleiden Institute for Genetics, Bioinformatics and Molecular Botany, Synthetic Biology of Photosynthetic Organisms, Dornburgerstrasse 159, 07743 Jena, Germany*

‡‡*Contributed equally*

¶¶*Corresponding Authors*

E-mail: F.BrancodosSantos@uva.nl; Nicolas.Schmelling@hhu.de

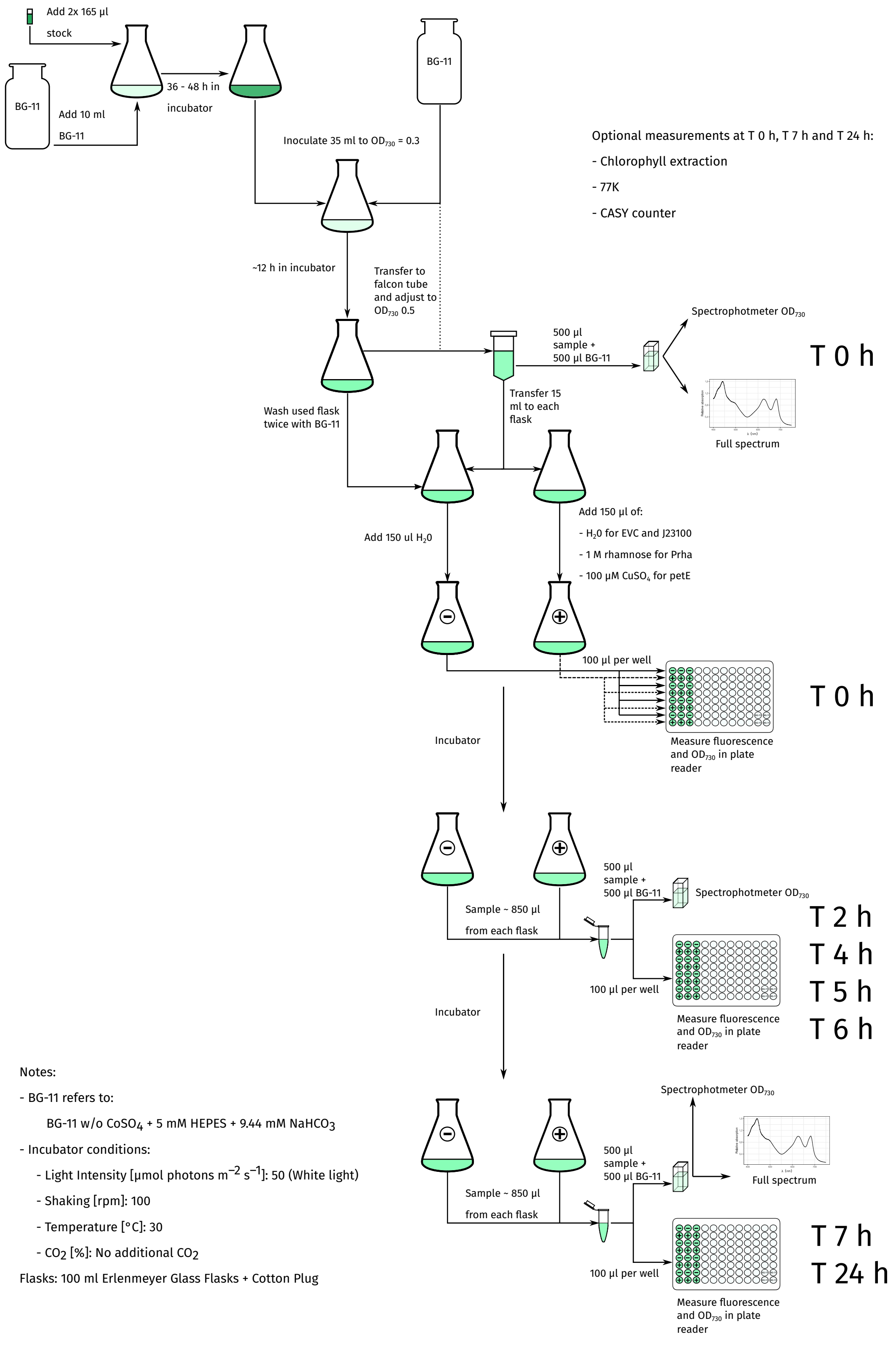

Figure S1: **Graphical protocol of promoter quantification assay.**

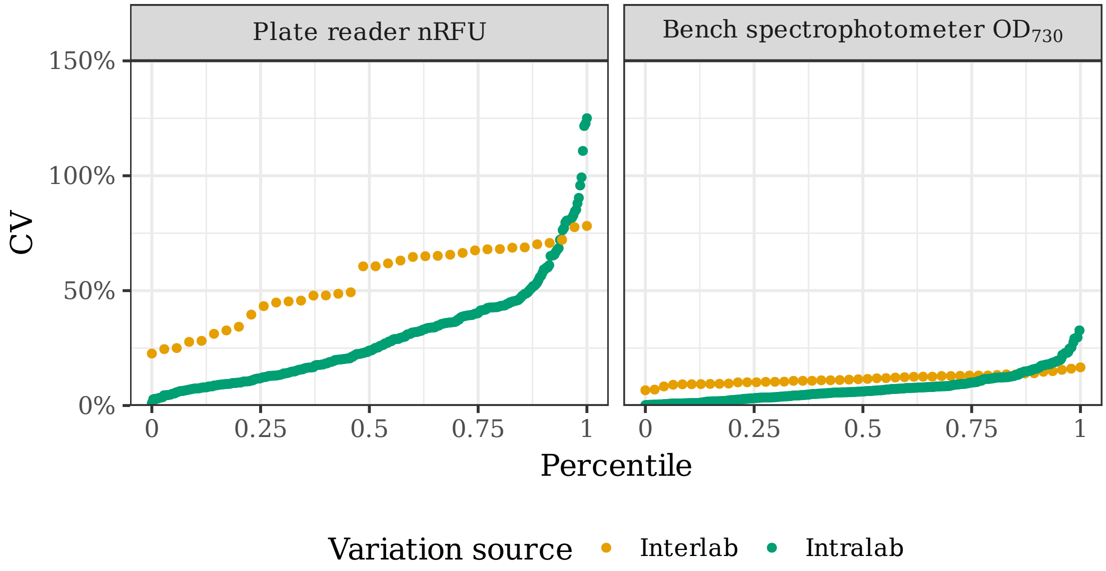

Figure S2: **Coefficient of variation (%) by percentiles for nRFU (left) and spectrophotometer OD_{730} (right) datasets.** The coefficient of variation was calculated for either all the replicates within a lab (intralab, in green) or for all the replicates across all labs (interlab, orange).

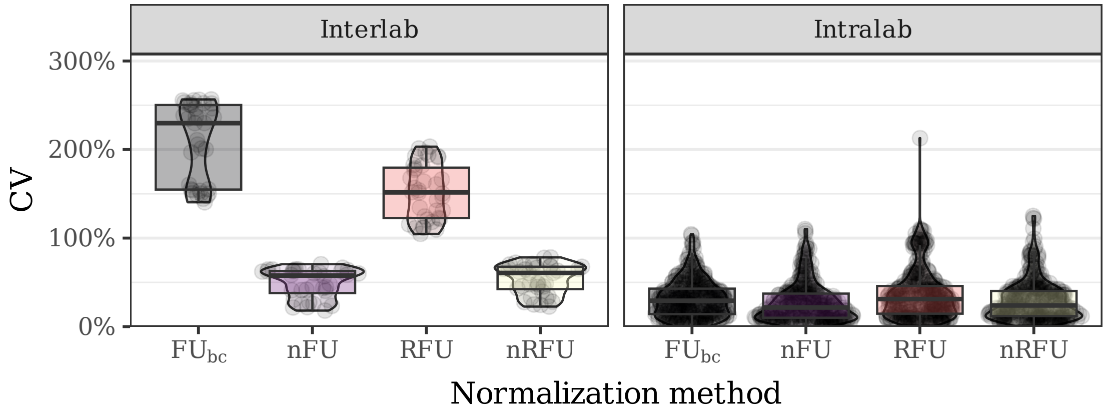

Figure S3: **Coefficient of variation (%) for different normalization methods at interlab (left) and intralab (right) level**. FU_{bc}: background-corrected fluorescence units. nFU: normalized fluorescence units. RFU: relative fluorescence units. nRFU: normalized fluorescence units. nFU was calculated with equation $nFU = \frac{FU_{bc}}{FU_{bc\ \ J23100-}}$. For the other variables, please see the Fluorescence analysis and normalization section in Materials and Methods.

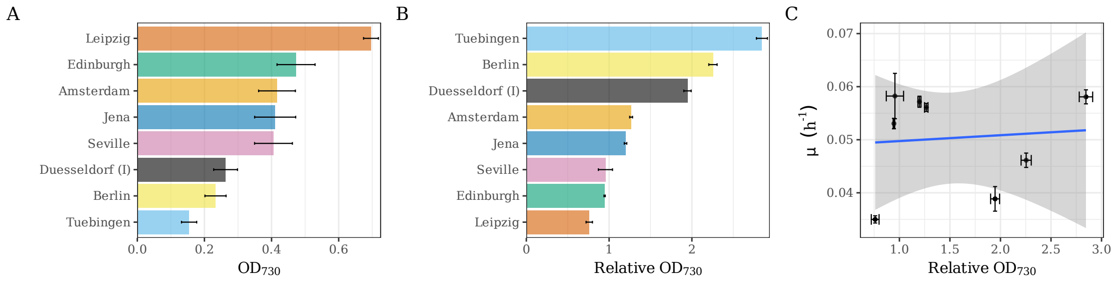

Figure S4: **Differences in spectrophotometer measurements across labs and its effect on growth rates.** A, OD_{730} values were recorded in each lab after measuring approximately the same concentration of cells. Bars show the average value, and error bars represent 95% CI (n = 4). B, relative OD_{730} values of initial measurements during the experimental assay. The recorded values at time 0h were divided by the measurements shown in panel A. Bars show the average value, and error bars represent 95% CI (n = 24 or 34). C, Correlation between estimated growth rates and relative OD_{730} from panel B. Points show the average growth rate and relative OD_{730}, horizontal error bars show the 95% CI of relative OD_{730}, and vertical error bars depict the 95% CI of growth rates. The blue line indicates the fitted linear model, and the shaded area shows the 95% CI.

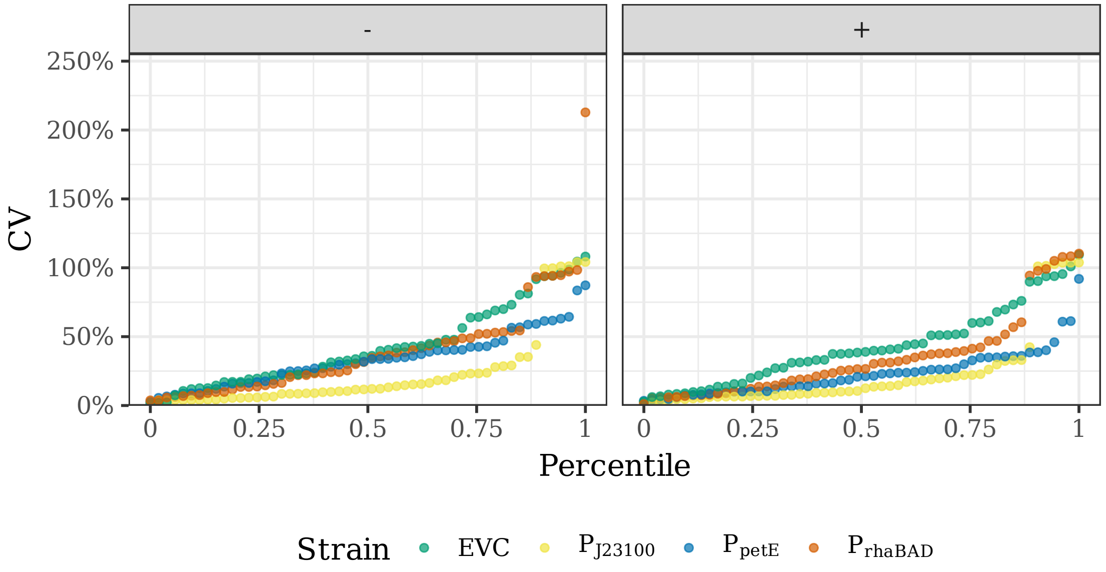

Figure S5: **Intralab coefficient of variation (%) by percentiles of RFU (not normalized) for uninduced (left) and induced (right) datasets.** The coefficient of variation was calculated for each strain (shown in different colors) based on all the replicates within a lab.

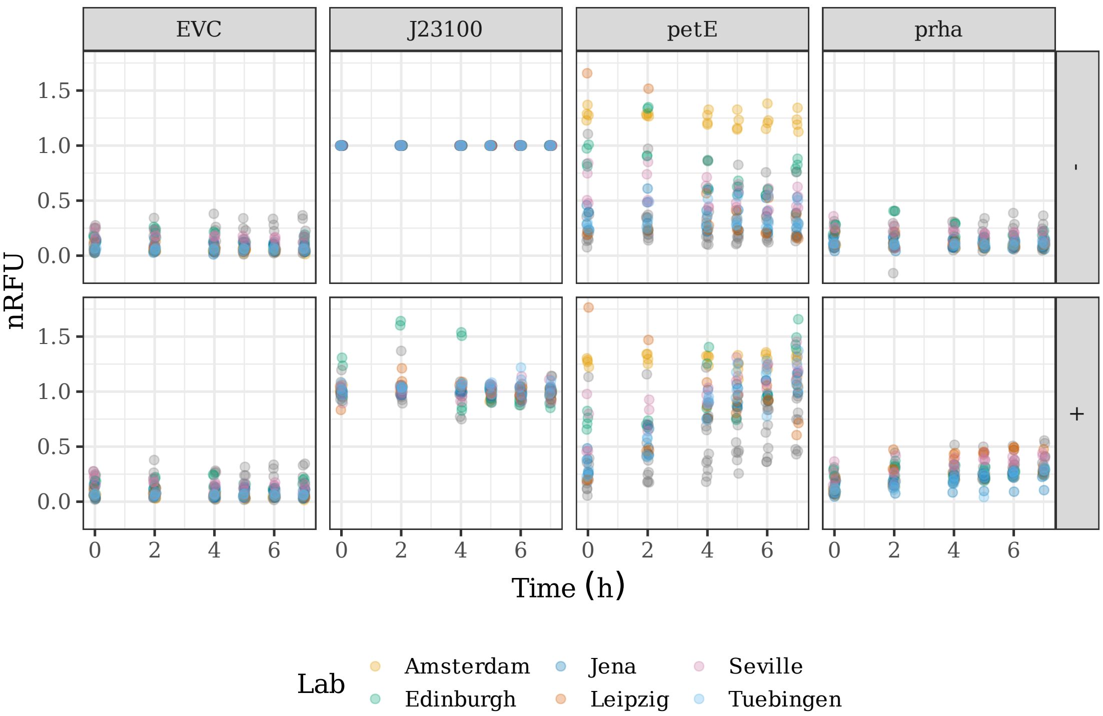

Figure S6: **Variation on time series of promoter assay.** The nRFU over time is shown for the four strains and two induction regimes, each depicted in an individual panel. The point's color indicates the lab where the measurement was performed.

Table S1: Reported fold changes of prha promoter variants in PCC 6803 in literature.

Publication	Promoter variant	Fold change
Kelly et al.[1]	Genomic integration, flanked by terminator sequences	3000x (estimated)
Behle et al.[2]	J23119 expressed RhaS	55x
Behle et al.[2]	J23111 expressed RhaS	39x
Liu et al.[3]	Plasmid based, including a RSW ribosome sequence	30x (estimated)
Kelly et al.[1]	Genomic integration, coexpressed with kanR	15x (estimated)
This study	J23100 expressed RhaS	5x

Table S2: Reported fold changes of petE promoter in PCC 6803 in literature.

Publication	Promoter/Measurement variant	Fold change
Briggs et al.[4]	WT, northern blot	no change
Giner-Lamia et al.[5]	Microarrays, 3 μm, 1h	63x
García-Cañas et al.[6]	RNAseq, 0.5 μm, 2h	25x
Giner-Lamia et al.[7]	WT, northern blot, 1 μm, 1h	6x
Englund et al.[8]	Expression from plasmid, fluorescence reporter	3x
This study	Expression from plasmid, fluorescence reporter	ranging from 2x to 6x

Preparation of BG11-PC

The following recipe was extracted from van Alphen et al..[9] For the purpose of this study, we removed copper from the recipe to be able to use the P_{petE} promoter as an inducible promoter.

Table S3: Composition of BG11-PC.

Compound	Concentration		Unit
	van Alphen et al.[9]	This study	
$NaNO_3$	1.5		$g \cdot L^{-1}$
K_2HPO_4	0.04		
$MgSO_4 \cdot 7H_2O$	0.075		
$FeCl_3 \cdot 6H_2O$	0.036		
$EDTA{-}Na_2 \cdot 2H_2O$	0.0186		
H_3BO_3	2.86		$mg \cdot L^{-1}$
$MnCl_2 \cdot 4H_2O$	1.81		
$ZnSO_4 \cdot 7H_2O$	0.222		
$Na_2MoO_4 \cdot 2H_2O$	0.391		
$CuSO_4 \cdot 5H_2O$	0.079	0	
$Co(NO_3)_2 \cdot 6H_2O$	0.049		

The original BG11-PC is separated into three stock solutions to avoid incompatible compounds (i.e. because of low solubility) at high concentrations (Table S3). The stock solutions for this medium are prepared as follows:

Stock 1

Dissolve $CaCl_2 \cdot 2H_2O$ in 700 mL ultra-pure H_2O in a glass measuring cylinder. Subsequently, add $NaNO_3$ and fully dissolve it by stirring the solution vigorously. Submerge the measuring cylinder in warm water to help dissolve $NaNO_3$ due to the endothermic nature of this process. Cool down the solution at room temperature once $NaNO_3$ is fully dissolved. Afterward, adjust to 1 L and filter sterilize the solution. The solution should be colorless in the end.

Stock 2

Dissolve $EDTA{-}Na_2 \cdot 2H_2O$ in a plastic measuring cylinder in 400 mL of purified water. Add $FeCl_3 \cdot 6H_2O$ and leave the mixture stirring overnight to dissolve all ingredients fully. On the next day, dissolve $MgSO_4 \cdot 7H_2O$ in 150 mL purified water in another measuring cylinder. Subsequently add the dissolved MgSO4 $\cdot 7H_2O$ to the $FeCl_3 \cdot 6H_2O$-$EDTA{-}Na_2 \cdot 2H_2O$ solution. Next, add 400 mL of Micronutrients solution (see below) and adjust to 1 L before filter sterilizing the final solution. In the end, the solution is greenish-yellow and should not discolor the filter. If it does, the solution should be remade.

Stock 3

Dissolve K_2HPO_4 in 1 L purified water and add $EDTA{-}Na_2 \cdot 2H_2O$. Subsequently, sterilize the solution by filtering. In the end, the solution should be colorless.

Micronutrients

Add the trace metal salts in the order listed in Table S4 to 1 L of purified water. Filter sterilize the resulting solution when all salts are fully dissolved. In the end, the solution should be colorless.

Medium Preparation

Prepare the final BG11 medium by adding 2.5 mL of each stock solution to 1 L of autoclaved purified water. For plates, add 15 g $\cdot$ L^{-1} agar to the desired volume of water, autoclave, and supplement the solution with stock 1, 2, and an EDTA-free stock 3. Optionally, prepare a 50-fold concentrated BG11 medium stock to simplify handling in small-scale applications (i.e., flask experiments). It is recommended to store the medium in a refrigerator to reduce the risk of contamination. Note that the solubility of EDTA at the low pH of stock 2 is too low to allow all EDTA to be added to stock 2. An equimolar concentration of EDTA and $FeCl_3$ ensures iron ions stay in solution in this acidic environment and allows for convenient preparation of plate-compatible BG11-PC.

Table S4: Composition of stock solutions used to prepare BG11-PC.

Stock solution	Compound	Concentration ($g \cdot L^{-1}$) van Alphen et al.[9]	This study
1	$NaNO_3$	600	
	$CaCl_2 \cdot 2H_{20}$	14.4	
2	$MgSO_4 \cdot 7H_2O$	30.0	
	$FeCl_3 \cdot 6H_2O$	1.62	
	$EDTA{-}Na_2 \cdot 2H_2O$	2.24	
	Micronutrients	400 mL	
3	K_2HPO_4	16	
	$EDTA{-}Na_2 \cdot 2H_2O$	5.2	
Micronutrients	H_3BO_3	2.86	
	$MnCl_2 \cdot 4H_2O$	1.81	
	$ZnSO_4 \cdot 7H_2O$	0.222	
	$Na_2MoO_4 \cdot 2H_2O$	0.391	
	$CuSO_4 \cdot 5H_2O$	0.079	0
	$Co(NO_3)_2 \cdot 6H_2O$	0.049	

Table S5: List of equipment used by each participant.

Participant	Incubator model	Spectrophotometer model	Plate reader model	Light source model
Amsterdam	New Brunswick Innova 44R	Biochrom PWA Lightwave II	BioTek Synergy Mx	Sylvania GRO-LUX F15T8/GRO/AQ
Berlin	Infors HT Multitron	Analytik Jena SPECORD 50 Plus	Tecan Spark Cyto	Osram Fluora L 36W/77
Duesseldorf I	Infors HT Multitron	Analytik Jena SPECORD 210 Plus	BMG labtech clariostar Plus	Osram Fluora L 36W/77
Duesseldorf II	New Brunswick Innova 42R	Analytik Jena SPECORD 210 Plus	BMG labtech clariostar Plus	Sylvania T8 Luxline Plus F 15W/865 G13
Edinburgh	PSI Algaetron 230, Infors Multitron	Biochrom PWA Lightwave II	BMG labtech FLUOstar Omega	Warm white LED (included with incubator)
Jena	PSI Algaetron 230	Eppendorf BioSpectrometer basic	Agilent BioTek Synergy H4 Hybrid Reader	Cool white LEDs with additional far-red LEDs at 735 nm (included in the incubator)
Leipzig	Infors Multitron and Minitron	Biochrom Libra S11	Tecan Infinite M200 Pro	Osram Fluora L 36W/77
Seville	Culture room and orbital shaker	JASCO V-650 for single measurements; Genesys 180 Thermo Scientific for spectral measurements	Thermo Fisher Varioscan Lux	Osram Substitube st8v 1.2m840-em 4000K
Tuebingen	New Brunswick Innova 2300	Analytik Jena SPECORD 50 Plus	Tecan Spark 10m Multi mode	Osram L 58W/954 LUMILUX de Luxe connected to REGIOLUX MLUD 1/58 EVG (230V-50 Hz)

References

(1) Kelly, C. L.; Taylor, G. M.; Hitchcock, A.; Torres-Méndez, A.; Heap, J. T. A Rhamnose-Inducible System for Precise and Temporal Control of Gene Expression in Cyanobacteria. *ACS Synthetic Biology* **2018**, *7*, 1056–1066.

(2) Behle, A.; Saake, P.; Germann, A. T.; Dienst, D.; Axmann, I. M. Comparative Dose–Response Analysis of Inducible Promoters in Cyanobacteria. *ACS Synthetic Biology* **2020**, *9*, 843–855.

(3) Liu, D.; Johnson, V. M.; Pakrasi, H. B. A Reversibly Induced CRISPRi System Targeting Photosystem II in the Cyanobacterium *Synechocystis* sp. PCC 6803. *ACS Synthetic Biology* **2020**, *9*, 1441–1449.

(4) Briggs, L. M.; Pecoraro, V. L.; McIntosh, L. Copper-induced expression, cloning, and regulatory studies of the plastocyanin gene from the cyanobacterium *Synechocystis* sp. PCC 6803. *Plant Molecular Biology* **1990**, *15*, 633–642.

(5) Giner-Lamia, J.; López-Maury, L.; Florencio, F. J. Global Transcriptional Profiles of the Copper Responses in the Cyanobacterium *Synechocystis* sp. PCC 6803. *PLOS ONE* **2014**, *9*, 1–16.

(6) García-Cañas, R.; Giner-Lamia, J.; Florencio, F. J.; López-Maury, L. A protease-mediated mechanism regulates the cytochrome c_6/plastocyanin switch in *Synechocystis* sp. PCC 6803. *Proceedings of the National Academy of Sciences* **2021**, *118*, e2017898118.

(7) Giner-Lamia, J.; López-Maury, L.; Florencio, F. J. Ni interferes in the Cu-regulated transcriptional switch petJ/petE in *Synechocystis* sp. PCC 6803. *FEBS Letters* **2016**, *590*, 3639–3648.

(8) Englund, E.; Liang, F.; Lindberg, P. Evaluation of promoters and ribosome binding

sites for biotechnological applications in the unicellular cyanobacterium *Synechocystis* sp. PCC 6803. *Scientific Reports* **2016**, *6*.

(9) van Alphen, P.; Abedini Najafabadi, H.; Branco dos Santos, F.; Hellingwerf, K. J. Increasing the Photoautotrophic Growth Rate of *Synechocystis* sp. PCC 6803 by Identifying the Limitations of Its Cultivation. *Biotechnology Journal* **2018**, *13*.
